# Society Meetings

**Published:** 1906-11

**Authors:** 


### SOCIETY MEETINGS.

The Roswell Park Medical Club of Buffalo at its recent annual meeting elected the following-named officers for the ensuing year: president, Arthur G. Bennett; vice-president, Prescott LeBreton; secretary-treasurer, George F. Cott.

The Homeopathic Medical Society of the State of New York held its semiannual meeting at Rochester, October 16, 1906, under the presidency of Dr. Newton W. Collins, of that city. The Western New York Homeopathic Medical Society held its regular quarterly meeting at the same time and conjointly with the state society.

The Buffalo Academy of Medicine at its regular meeting held September 25, 1906, elected the following-named physicians, all residing in Buffalo, to membership:

Drs. Clifford R. Orr, Hyatt Regester, George U. McMichael, Charles A. Wall, Bernard Cohen, Nelson W. Wilson, Arthur Eisbein, Charles P. Eller, John S. McFarland, Roland O. Meisenbach, E. A. Southall, Frank H. Ransom, Jr., Frederick J. Parmenter, William B. May, Charles E. Abbott, A. E. Sohmer, J. B. Young, James E. King, Edwin R. Gould, John D. Bonnar, H. B. Brownell, J. W. Fitzgerald, Thomas H. McKee, Albert

J. Harris, Frederick Zingsheim, James P. Barr, James J. McFadden, John D. Howland, L. E. Villiaume, Frank A. Harrington, Hugh McIntyre, James H. Lewis, Abram L. Weil, Robert F. Sheehan, Jr., Albert W. Palmer, Alexander Allen, and William C. Lewin.

This increases the roll of the Academy to about two hundred and fifty members.

The Academy held meetings during the month of October as follows:

*Section on Surgery.*—Tuesday, October 2, 1906, at 8:30 P. M. Program: (a) Results of some intracranial operations with exhibitions of cases, George F. Cott; (b) Chronic urethritis and an improved method of applying medication to the urethra, James A. Gardner.

*Section on Medicine.*—Tuesday, October 9, 1906, at 8:30 P. M. Program: (a) An important factor in the causation and treatment of many so-called functional disorders, Carl G. Leo-Wolf, Niagara Falls, N. Y.; (b) The differential diagnosis of the so-called rheumatoid diseases, Roland O. Meisenbach.

*Section on Obstetrics and Gynecology.*—Tuesday, October 23, 1906, at 8:30 P. M. Program: (a) Some further observations on why minor gynecological operations fail to give results, Sigmund Goldberg; (b) Gonorrhea in the pregnant female, Cornelius J. Carr.

